# Relative geographic range of sibling species of host damselflies does not reliably predict differential parasitism by water mites

**DOI:** 10.1186/1472-6785-13-50

**Published:** 2013-12-18

**Authors:** Julia J Mlynarek, Wayne Knee, Mark R Forbes

**Affiliations:** 1 Department of Biology, Carleton University, Nesbitt Building, 1125 Colonel By Drive, Ottawa, ON K1S 5B6, Canada; 2 Canadian National Collection of Insects, Arachnids and Nematodes, Agriculture and Agri-Food Canada, 960 Carling Avenue, K.W. Neatby Building, Ottawa, ON K1A 0C6, Canada

## Abstract

**Background:**

One of the main challenges in evolutionary parasitology is to determine the factors that explain variation among host species in parasitism. In this study, we addressed whether host phylogeny or ecology was important in determining host species use by water mites. Parasitism (prevalence and intensity) by *Arrenurus* water mites was examined in relation to geographic distribution of host damselflies from sibling species pairs. In addition, the likelihood of putative mite species parasitizing both species of a host species pair was explored.

**Results:**

A total of 1162 damselflies were examined for water mites across four sites in Southeastern Ontario. These damselflies represent ten species (five closely related host species pairs) in the Coenagrionidae. Only two of the five species pairs showed near significant or significant differences in prevalence of infection by mites. In one of those species comparisons, it was the less widespread host that had higher water mite prevalence and in the other species comparison, the less widespread host species had lower water mite prevalence. Only one of the five pairs showed a significant difference in intensity of infection; intensity was higher in the species with a smaller geographic distribution. Based on the COI barcode, there were nine water mite clades (OTU) infecting these ten host species. Three *Arrenurus* OTUs may be host monospecific, four OTUs were specific to a given host species pair, and two OTUs infected at least three host species. Host species in each species pairs tend to share at least one of the *Arrenurus* OTU. No striking differences in mite species diversity were found among species in any species pair. Finally, the *Arrenurus* examined in this study appear to be ecological specialists, restricted to a particular type of habitat, parasitizing few to many of the host species present in that site or habitat.

**Conclusions:**

Although differences in levels of parasitism by water mites exist for some closely related hosts species, no such differences were found between other related host species. Differences in geographic range of related host species does not reliably explain differential levels of parasitism by water mites.

## Background

A major challenge of the combined fields of ecological and evolutionary parasitology is to understand the determinants of variation in parasitism between closely related host species [1,2]. Some of the proposed determinants include size of a host species’ geographic distribution [3], host size [4], taxonomic relatedness of host species [5] and habitat requirements of both hosts and parasites [6,7]. Most of the research in this area has been done on vertebrate hosts (e.g., [8]). A question remains as to whether it is possible to extrapolate findings of studies on vertebrates and their parasites to similar problems with invertebrate host species and their parasites.

Some generalities can be drawn from studies on parasitism of vertebrates as a guide to explorations of parasitism of invertebrate hosts. For example, host species with a greater geographic range may come into contact with infective stages of more parasite species and therefore have higher diversity of parasites or higher parasitism levels [9,10]. Alternatively, hosts with larger geographic areas might evolve more generalized immune responses and therefore have fewer parasites in local areas (*cf.*[11]). Using damselflies and gregarines, Mlynarek et al. [11] demonstrated that in three out of seven species pairs, one host species had a statistically higher prevalence of gregarines, when controlling for collecting site. In two of those species comparisons, the sibling species with the smaller geographic distribution had higher prevalence; in the latter comparison, the species with the larger geographic distribution had the higher prevalence. Similarly, in two out of seven species pairs, the species that had a smaller geographic distribution had a statistically higher gregarine intensity [11].

Other studies with vertebrates and their parasites have demonstrated that host geographic distribution size can be related to parasite species richness [3,4,12]. The recurrent relation between host geographical range and parasite species diversity has been explained in part by increased chance of intense localized encounters between interacting species with the increasing range size of the host species [10,13]. Although host species with larger geographic distributions are expected to have more parasite species than closely related host species with smaller geographic ranges [10], it is unknown whether such patterns are observed often at given sites. Species with larger geographical ranges typically occupy more sites and might be subjected to more studies: a potential problem recognized early [12].

Several studies have looked at relationships between host and/or habitat characteristics and parasitism levels in insect host-invertebrate parasite associations. Durrer and Schmid-Hempel [14] studied regional and local abundance of bumblebees with respect to external and internal parasite load and diversity. Those researchers found that there was a positive correlation with parasite load and colony size, and also that parasite diversity correlated positively with regional distribution of hosts, but host body size did not affect any of the measures. Jaenike and Perlman [15] reviewed the role of nematode parasites on mycophagous *Drosophila* behaviour, reproduction and community structure. Their work demonstrated that parasitism facilitates co-existence between closely related hosts and reduces fertility in female *Drosophila*. One other study compares parasitism levels explicitly between species differing in geographical representation, but there the focus is on gregarine parasites of calopterygid damselflies in allopatric versus sympatric associations [16].

For various reasons, the extent to which parasitism and parasite diversity varies as a function of geographic range can be explored effectively using coenagrionid damselflies and *Arrenurus* water mites. First, there is interspecific variation in ecological and evolutionary traits in closely related damselfly host species (e.g., host geographical distribution, local abundance, see [17]). Second, it is known that there are several generalist species among mites [18]. Third, *Arrenurus* infection has been demonstrated to vary between closely related lestid host species in different habitats [19] and between more distantly related host species in the same habitat [20]. Fourth, *Arrenurus* water mites first parasitize teneral adults and are easily recognized as unengorged but live, unengorged and resisted, and engorged external parasites. Importantly, these mites remain on one host for the duration of their parasitic life stage [21]. Larval *Arrenurus* embed their mouthparts into the teneral cuticle of their host and secrete a stylostome or feeding tube. Mites remain usually on the ventral side of the thorax, between the legs, or the last abdominal segments [21]. Once the host returns to a water body to mate and/or lay eggs, the water mite detaches and falls into the water to continue its life cycle. Different species of water mite are also known to inhabit different types of water bodies [21], setting the stage for particular associations to be habitat specific.

The objectives of this study were several-fold. First, we tested whether closely-related host species were differentially infected by water mites. We then determined if the relative size of geographical distribution of sibling host species was associated with relative parasitism levels and moreover whether the occasional patterns observed between gregarines and damselflies documented in Mlynarek et al. [11] was also observed when considering ectoparasitic mites. More specifically, we were testing whether host species with smaller geographic distribution have higher levels of ectoparasitism, by analogy. We then assessed whether closely-related host species were infected by the same putative *Arrenurus* species using molecular techniques for parasite identification thereby making comparisons of parasitism between sibling host species more direct. Related to this objective, we were interested in whether the host species with higher measures of parasitism were also those with greater numbers of parasite species. In addressing these objectives, we also examined the extent to which *Arrenurus* water mite species show habitat or host species preferences. In summary, we were interested in whether a host species geographical distribution influenced measures of ectoparasitism and whether the patterns were repeatable or potentially affected by moderator variables (host species identity, habitat used).

## Results

### Measures of general *Arrenurus* parasitism

In total, 1162 damselflies were collected across four sites in Southeastern Ontario, within a 20-km radius of the Queen’s University Biological Station (Table 1).

**Table 1:** Details of parasitism in ten species of damselflies sampled at four sites

**Host species**	**Location**	**N (N infected)**	**Geographic range (10**^**6 **^**Km**^**2**^**)**	**Mite OTU**	**Prevalence (95% CI)**	**Intensity (95% CI)**
*Argia moesta*	Lake Opinicon	90 (4)	4.10	1, 7	0.04 (0.01–0.11)	1.50 (1–1.75)
*A. violaceae*	Lake Opinicon	97 (8)	3.98	1, 9	0.08 (0.04–0.16)	5.75 (1.38–18.13)
*Enallagma boreale*	Barb Marsh	57 (15)	6.53	4, 5	0.26 (0.16–0.39)	4.60 (2.86–7.36)
*E. ebrium*	Barb Marsh	106 (31)	4.35	4, 5	0.29 (0.21–0.39)	10.16 (6.81–13.68)
*E. signatum*	Lake Opinicon	120 (32)	3.23	1, 2	0.27 (0.19–0.36)	9.81 (5.16–22.09)
*E. vesperum*	Lake Opinicon	101 (54)	2.6	1	0.54 (0.43–0.63)	11.35 (7.00–18.87)
*Ischnura posita*	Osprey Marsh	77 (4)	3.13	3, 4, 6	0.05 (0.01–0.13)	3.25 (1.00–5.50)
*I.verticalis*	Osprey Marsh	23 (4)	4.36	1, 3, 6	0.17 (0.05–0.39)	6.75 (1.50–16.50)
*Nehalennia gracilis*	Hebert Bog	208 (25)	1.51	8	0.12 (0.08–0.17)	2.12 (1.60–3.44)
*N. irene*	Hebert Bog	284 (45)	4.03	8	0.16 (0.12–0.21)	1.78 (1.42–2.76)

N represents the number of individuals of each species sampled. Geographic range of the host is provided. Confidence limits for prevalence and intensity are shown respectively.

**Figure 1:**
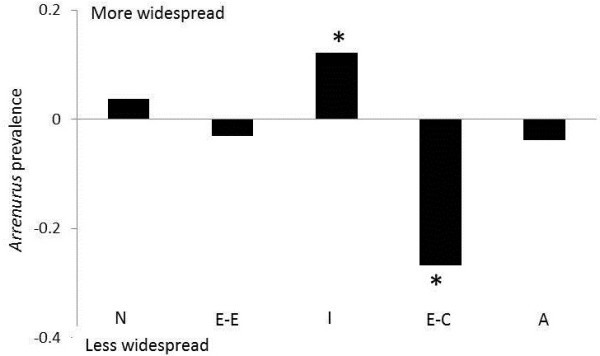
**Within species pair difference in measures of prevalence between species.** A positive difference means that the more widespread species of the species pair has higher estimate of prevalence of infection (from Table 1); a negative difference means that the less widespread species has higher estimate of prevalence. Actual significant differences in prevalence values are marked by *. The organization of the species pairs reflects geographical distribution differences within the species pair from most different to least different. Key: A, *Argia*; C, *Calopteryx*; E-C, *Enallagma Chromatallagma*; E-E, *Enallagma Enallagma*; I, *Ischnura;* N, *Nehalennia.*

Prevalence of *Arrenurus* varied considerably between host species and species pairs (Table 1). Overall prevalence varied between 4% in *Argia moesta* and 54% in *Enallagma vesperum.* Both of those species were collected from Lake Opinicon. Significant or near significant differences in prevalence between species in sibling species pairs were found in two of five comparisons. In particular*,* there was a significant difference in prevalence between the species of *Enallagma* (*Chromatallagma*) (χ^2^ = 16.57 df = 1 p < 0.001) where 54% of *Enallagma vesperum* individuals were infected compared to 27% of *E. signatum. Ischnura posita* had a prevalence of 5% whereas *I. verticalis* had a prevalence of 17%. The difference in prevalence in this species pair was close to significant (χ^2^ = 3.58 df = 1 p = 0.06; Figure 1). In comparison, no significant differences in prevalence were found for *Argia, Enallagma* subgenus *Enallagma* and *Nehalennia* (Table 2).

**Table 2:** Results of the Chi-square test for comparing prevalence and Bootstrap t-test for mean intensity of parasitic infection within the five Zygoptera species pairs

**Host species pair**	**Prevalence**			**Intensity**	
	**χ**^**2**^	**df**	**p**	**t-value**	**Bootstrap p**
*Argia*	1.12	1	0.29	1.05	0.41
*Enallagma E*	0.16	1	0.69	2.65	**0.01**
*Enallagma C*	16.57	1	**<0.01**	0.33	0.35
*Ischnura*	3.58	1	**0.06**	0.66	0.54
*Nehalennia*	1.44	1	0.52	0.70	0.51

**Figure 2:**
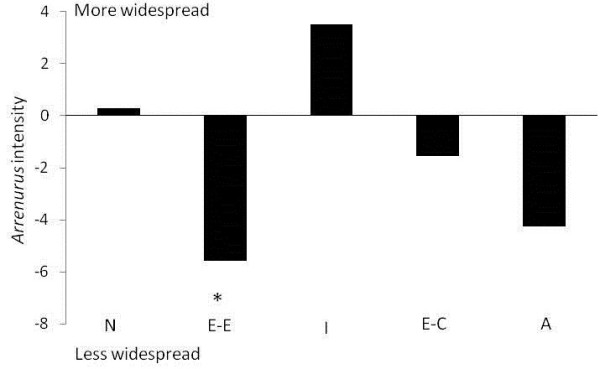
**Within species pair difference in measures of intensity between species.** A positive difference means that the more widespread species of the species pair has higher estimate of intensity of infection (from Table 1); a negative difference means that the less widespread species has higher estimate of intensity. Actual significant differences in prevalence values are marked by *. The organization of the species pairs as in Figure 1*.*

The data on mean intensity of infection told a similar story. Overall, the minimum mean intensity was 1.5 in *A. moesta* and the maximum was 11.9 in *Enallagma ebrium* (Table 1). In *Enallagma* (*Enallagma), Enallagma boreale* was infected by an average of 4.6 mites per infected host individual, but *E. ebrium* was infected by an average of 10.2 mites per individual (t = −2.65 bootstrap p = 0.01; Figure 2). In *Argia, Enallagma* (*Chromatallagma), Ischnura* and *Nehalennia* no significant differences in intensity were found between the species (Table 2).

At least two species pairs showed no differences in either prevalence or mean intensity of infection and yet sibling species differed in geographical distribution (*Argia* and *Nehalennia*). The other three species pairs had a significant or near significant difference in prevalence or a significant difference in intensity. In *Enallagma* (*Chromatallagma*), *E. vesperum* had significantly higher prevalence of parasitism: this species has the smaller geographic distribution of the pair (2.60 x 10^6^ km^2^ compared to 3.26 x 10^6^ km^2^ in *E. signatum;* Table 1). Similarly, in *Enallagma* (*Enallagma*)*, E. ebrium* had significantly higher intensity of parasitism; *E. ebrium* is less widely distributed (4.35 x10^6^ km^2^) than *E. boreale* (6.53x10^6^ km^2^; Table 1). In *Ischnura*, *I. verticalis* has both nearly significant higher prevalence of water mite infection and larger geographic distribution than *I. posita* (4.36 x 10^6^ km^2^ compared to 3.13 x 10^6^ km^2^; Table 1). Thus in 60% of cases, sibling host species with differences in size of geographic distribution differed in either prevalence or intensity of parasitism, but in those cases the actual differences are inconsistently related to relative size of the pair member’s geographic distribution.

### DNA barcoding

COI was amplified from 62 water mites, with 587 characters in total, 296 constant, 50 parsimony-uninformative and 241 parsimony-informative. Mean base pair frequencies (A: 0.31574, C: 0.21282, G: 0.13819, T: 0.33325) were found to be homogenous across all specimens (χ^2^ = 52.66, df = 207 *P* = 1.0).

**Figure 3:**
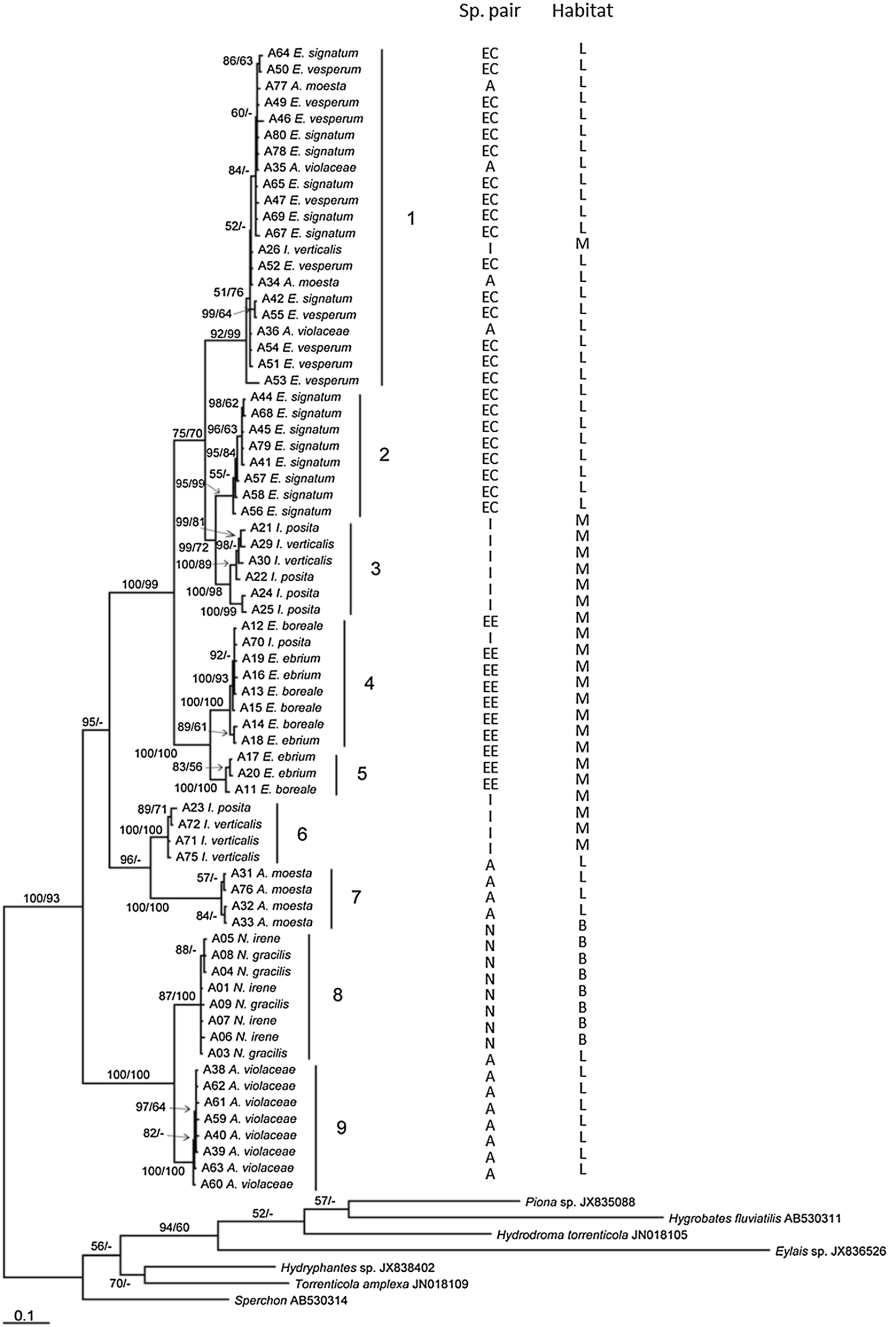
**Majority rule consensus tree of 19802 trees generated by Bayesian MCMC analysis (10 million generations) of 587 bp fragment of COI from 77 water mites, 70 ingroup and 7 outgroup specimens (TL = 1048, CI = 0.4599, RI = 0.8617) posterior probability >50% / jackknife support.** Species pair A = *Argia;* EE = *Enallagma (Enallagma);* EC = *Enallagma (Chromatallagma);* I = *Ischnura;* N = *Nehalennia*, habitat type B = Bog; L = Lake, M = Marsh.

Bayesian inference (BI) of the COI dataset was performed for 10 million generations, producing 19802 trees (after burn-in), which were summarized in a majority rule consensus tree (TL = 1048, CI = 0.4599, RI = 0.8617) (Figure 3). The BI consensus tree was well supported, with most nodes having moderate to high posterior probabilities and jackknife support (Figure 3). The ingroup are divided into nine well-supported clades, hereafter referred to as OTU (Operational Taxonomic Units). Average interspecies divergence was 15% ±1.1 (5–21%), and average intraspecies divergence was 0.7% ±0.3 (0–4%). Average divergence between OTU 1, 2 and 3 was 9% ±1, divergence between OTU 4 and 5 was 6% ±0.3, between 6 and 7 was 10% ±0.3, and species 8 and 9 show 8% ±0.3 divergence. Considering the high level of divergence between OTU and the low divergence within each OTU, as well as the strong support for each OTU in the phylogenetic reconstruction, it appears that each OTU may be distinct. However, we do not advocate the notion that these are new species, distinguishing between species using pairwise distances from a fragment of a single gene is not a reliable taxonomic approach [22].

**Figure 4:**
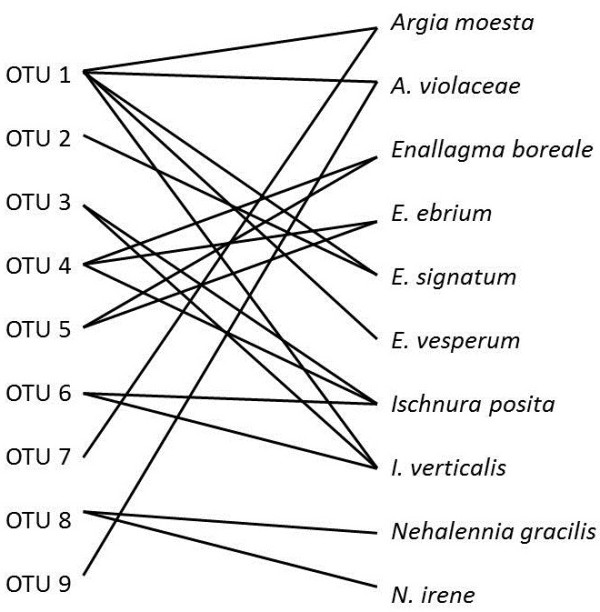
**Schematic network of damselfly-water mite OTUs.** The solid lines represent associations between a damselfly host species and a water mite OTU.

Based on our Bayesian tree (Figure 3), we found in *Argia* that there are three *Arrenurus* OTUs that infect *Argia moesta* and *A. violaceae*. OTU 9 is specific to *A. violaceae* and OTU 7 to *A. moesta* (Figure 3). Each of these OTUs is associated with a single host species, therefore we consider them to be candidates for specialist parasites. OTU 1 was collected both *A. moesta* and *A. violaceae*, and also two other *Enallagma* species (*E. signatum* and *E. vesperum*) from lake Opinicon indicating that this mite has a broad host species spectra (Figure 4). In addition, there is one record of this mite OTU present on *Ischnura verticalis* in a marsh habitat. Interestingly, this broad host generalist was the only OTU collected from *E. vesperum* (out of 5 mites sampled). Thus, we discovered that mites of two host species pairs (examined first) shared a mite species (Figure 3).

Similarly, the two *Enallagma* (*Enallagma*) host species were both infected by two closely related OTUs (4 & 5)*.* These OTUs are distinct, based on sequence divergence and branch support, but are closely related to one another (Figure 3). Therefore these two OTUs were collected from at least these two species of damselflies. However, we could not determine whether these two parasite species were present on other host species. Again, it appears that sibling species share mite species. *Ischnura* is an interesting species pair in that four different OTUs have been collected from these hosts, despite the limited sampling. OTU 3 and 6 were collected from both *Ischnura* species again supporting sharing of parasites. But OTU 1, the aforementioned generalist on damselfly host species in the lake, was found on one *Ischnura verticalis* individual from a marsh inlet site. Additionally, OTU 4 (Figure 3) infecting *I. posita* is shared with *Enallagma* (*Enallagma*) damselfly hosts from a different marsh. Finally, both *Nehalennia* species collected in the bog are infected by OTU 8 [17]. The results show that the sharing of mite species by sibling species of damselflies (and beyond those host clades) is a common occurrence (Figure 4). Species richness and composition within the host species pair did not differ (range of Bray-Curtis dissimilarity Index varied between 0.00 in *Nehalennia* to 0.86 in *Argia*) as much as between species pairs where the Bray-Curtis dissimilarity index ranged between 0.79-1.00. The dissimilarity index on 0.79 was determined between *A. violaceae* and *E. vesperum,* which share water mite OTUs (Figure 4). Figure 3 is also annotated with whether or not the host and mites were collected from a lake, marsh or bog. It appears that mite species are restricted to one type of habitat but this is not a “hard and fast” rule.

## Discussion

Relative geographic distribution size did predict relative prevalence of parasitism in some species pairs, but not reliably across all species pairs. To reiterate, there were significant differences in prevalence for *Enallagma* (*Chromatallagma*) species pair where the less widespread species had a higher prevalence of *Arrenurus* spp. In one instance (*Ischnura* species pair) did the more widespread host species, *I. verticalis*, have significantly higher intensity of *Arrenurus* than the more geographically restricted host species *I. posita*. Even referring to these two comparisons, geographic distribution is not a reliable predictor of parasitism in these types of insect host-invertebrate parasite associations. Across all other species pairs, differences in geographic distribution were evident, but no species differences is relative prevalence of ectoparasitism was observed. And furthermore, in only in the *Enallagma* (*Enallagma*) species pair, did the less widespread species have a higher mean intensity of infection by *Arrenurus* than the more widespread *Enallagma* species. Across all other species pairs, there were no species differences in mean intensity of mite parasitism.

In comparison to the findings in this study, Tella et al. [9] tested other factors that could potentially mask the effect of host geographic distribution such as sampling effort, vector availability and embryonic development time, but the researchers concluded that hematozoan prevalence appears determined by host geographic distribution. Mlynarek et al. [11] also reported that measures of gregarine parasitism were higher in the less widespread species pair in four out of the seven damselfly species pairs. Additional comparisons with this study demonstrate that the species pairs respond differently, depending on the type of parasite studied. For example, in Mlynarek et al. [11]*Ischnura posita* had a significantly higher prevalence of gregarines than its more widespread counterpart, *Ischnura verticalis*. In this study, *Ischnura verticalis* has a near significant higher prevalence of *Arrenurus* water mites than *I. posita*. Relative size of a host’s geographic distribution, therefore, can have different or no effect on relative parasitism, depending on the type of parasite studied.

Based further on Mlynarek et al. [11] and this study, 12 species comparisons were done with respect to prevalence or intensity of either gregarines or mites. In half of the 12 species pair comparisons, there were statistical differences between species in either or both prevalence and intensity of either gregarine or mite infections. In four of these cases, the less widespread host species had higher prevalence and/or intensity of the parasite of interest whereas in the two remaining cases, it was the more widely distributed host with the higher parasite levels (prevalence of gregarines for *Nehalennia* and prevalence of mites for *Ischnura*). In one case, *Enallagma (Chromatallagma)*, there were significant differences in both prevalence of mites and prevalence of gregarines whereby the less widespread host had higher prevalence of parasites. Stated another way, in four (57%) of the seven comparisons involving gregarines and in three (60%) of the five comparisons involving mites, closely related host species differed in one or more measures of parasitism. In the remaining 40% of the cases, the closely related species did not differ in measures of parasitism despite showing differences in geographical range. Further, in one (25%) of four comparisons involving significant differences in gregarines, it was the more widely distributed host that had higher gregarine prevalence. A similar result was found where one (33%) of three comparisons (*Ischnura*) involving significant differences in mite intensities, was attributed to the species with the greater geographic range.

In this study, using COI barcodes, there are nine OTUs within *Arrenurus* infecting the ten damselfly species. *Arrenurus* OTU 2, 7 and 9 appear specialized on a specific damselfly host species (*E. signatum, A. moesta* and *A. violaceae*), but sampling was limited. OTU 1 infects four host species in lakes. The *Arrenurus* OTUs 3,4,5,6 and 8, collected in the Marsh and bog, infect at least two species each. Water mites appear affiliated with a habitat and not so much specialized on phylogenetically related host species. Similarly to Krasnov et al. [7], habitat was a strong factor influencing flea parasitism on rodents in the Negev desert. As well, in our case and that of Krasnov et al. [5], both host species’ and habitat characteristics are important in determining parasite species composition. Except for OTU 1, collected from an *I. verticalis* host, all the water mites are restricted to one particular habitat type. A potential reason could be that this *I. verticalis* individual was a vagrant into the marsh where it was collected, or that this mite is found in many habitats, mostly in lakes and rarely in others. The important point is that comparisons of parasitism between sibling species based on broad taxonomic identification of the parasites is somewhat permitted because all sibling host species share at least one mite species. If there was some strong interaction between a host geographic range and the intensity of interactions with given parasite species, it should be detected using even broad taxonomic comparisons, where only mites are being compared and not every parasite is identified to species.

There is a different pattern when comparing a group of parasites without considering broad taxonomic identification. When considering parasite species richness, it is expected that the more widespread hosts have a higher richness of parasite species [3]. In cricetid rodents, host geographic distribution was strongly associated with *Demodex* mite species richness [3]. In waterfowl, Gregory [12] demonstrated a clear positive relationship between host geographic range and parasite species richness, using cross-species comparisons. In European fresh-water fish, Simková et al. [4] concluded that host geographic distribution influenced parasite species richness through its effect on host local abundance and occurrence. Their research was based on 39 fish host species and a comprehensive assessment of internal and external parasites. In our case, we did not see this occurring but only can report on nine mite species across five pairs of damselfly host species. Notwithstanding, host species in each pair have a comparable number of mite species infesting them, even though certain host species might have species-specific parasites. The only exception in our study was *Enallagma* (*Chromatallagma*) species pair where the more widespread *E. signatum* has two *Arrenurus* OTUs (one host specific) whereas *E. vesperum,* the more restricted species, is only infected by one *Arrenurus* OTU. Even here, the conclusion that one host species has a higher parasite species diversity has to be met with scepticism, especially given that, when controlling for site, only one out of six instances does the more widespread species show this tendency toward higher parasite species richness.

## Conclusions

In this study, we documented host- parasite associations in multiple species pairs. We observed that there appear to be varying degrees of specificity in host range of *Arrenurus* mites. In conjunction with the findings of Mlynarek et al. [11] on internal parasites of damselflies, our findings suggest that host species’ geographic distribution size does not reliably explain relative species differences in measures of parasitism. Water mites species found in one type of habitat tend not to be present in another type of habitat but can parasitize few to many host species found in a habitat. More work is needed to further elucidate *Arrenurus* species boundaries, using additional molecular markers or rearing larvae for adult identification. Further work is needed on determining host species use before we can test the importance of other ecological factors (e.g. phenology or regional representation of both hosts and parasites) on past and ongoing evolution of these associations.

## Methods

### Specimen collection

Ten species of Coenagrionidae (*Argia moesta and A. violaceae; Enallagma boreale and E. ebrium; E. signatum and E. vesperum; Ischnura posita and I. verticalis; and, Nehalennia gracilis* and *N. irene*) were collected belonging to five sibling species pairs in Coenagrionidae based on (sub)generic affiliations (*Argia*, *Enallagma* (*Enallagma*), *Enallagma* (*Chromatallagma*) subgenus, *Ischnura* and *Nehalennia*). We considered the two *Enallagma* species pairs as separate because they are considered to be in separate subgenera [23]. The particular host species were chosen because a second closely related host species was found in sympatry and because species in sibling species pairs differed in geographic distribution size (minimum of 100 000 km^2^ difference between the two species in each pair, see [11]).

We collected adult damselflies using aerial sweep nets between 17 May and 15 July 2010 and 30 May and 15 July 2011. Damselflies in each species pair were collected from the same site at the same time (see below). The damselflies were stored in separate vials with 95% ethanol.

Species pairs were collected at different sites and times because host emergence varies temporally and spatially. *Argia moesta* and *A. violaceae* and *E.* (*Chromatallagma*) *signatum* and *E.* (*Chromatallagma*) *vesperum* were collected at the edge of Lake Opinicon (44°33’56.32”N, 76°19’26.46”W) on 30 June to 10 July 2010; the species *E.* (*Enallagma*) *boreale* and *E.* (*Enallagma*) *ebrium* were collected at Barb’s Marsh (44°31’27.54”N, 76°22’25.89”W) on 25 May, from 7 to 10 June 2010 and from 31 May to 21 June 2011, *I. posita* and *I. verticalis* were collected at a slow stream by Osprey Marsh (44°30’43.74”N, 76°23’39.32”W) on 4 and 10 July 2010, *N. gracilis* and *N. irene* were collected at Hebert Bog (44°29’54.69”N, 76°24’ 53.66”W) on 7 and 30 June 2011 and from 6 June to 18 July 2011.

All individuals collected were inspected for parasitism by looking at ventral side of the thorax and abdomen. All *Arrenurus* spp. water mites were counted.

### Statistical measures of general parasitism

Differences between sibling species in prevalence, the proportion of hosts infected with at least one larval *Arrenurus*, and intensity, the mean number of *Arrenurus* on only the infected hosts, was assessed in QP3.0 [24]. Prevalence estimates were provided with Clopper-Pearson 95% confidence intervals [25] whereas mean intensity estimates were provided with bootstrap confidence limits [24].

More specifically, we compared difference in prevalence between species in each species pair using the chi-square test. To test for differences in intensity, we performed a Bootstrap 2-sample t-test (with 2000 bootstrap replicates) between species in each species pair. We, therefore, performed ten tests, five tests to test for differences in prevalence between species in five species pairs and five tests to test for differences in mean intensities between species in the five species pairs. Both series of statistical tests were done in QP3.0 [24].

To explore any potential effects of geographical distribution on parasite prevalence or intensity, we simply inspected whether any significant differences between species in species pairs seemed to be predicted reliably by either the more or less widely distributed host species having either the higher or lower prevalence or intensity of *Arrenurus* spp. infection.

### DNA barcoding of *Arrenurus*

To address whether host species of sibling species pairs might share putative mite species, and to have a preliminary account of host species range of those mite “species”, DNA extractions from 62 larval mites was performed (from 70 larval water mites selected haphazardly, based principally on host species and site of host collection). This haphazard selection was as follows: five water mites were obtained from each host species from three different host individuals with mites coming from the thorax and/or abdomen (because thoracic and abdominal mites are often different species based on laboratory rearing of larval water mites through to adulthood (Bruce Smith, pers. comm.). All ten host species had thoracic mites. Four of ten species also had abdominal mites. In those latter species, both thoracic and abdominal water mites were collected, which is why sample size was initially 70.

Total genomic DNA was extracted from whole specimens for 24 hours using a DNeasy Tissue kit (Qiagen Inc., Santa Clara, CA, USA). Following extraction, mites were removed from the extraction buffer, and genomic DNA was purified following the DNeasy Tissue kit protocol. PCR amplifications were performed following the protocol of Mlynarek et al. [17], amplification cycles were performed on an Eppendorf ep Gradient S Mastercycler (Eppendorf AG, Hamburg, Germany). Primer pairs Alt-ALF1 (5′–GCDTGRTCWGGRATAGTDGGAGCMAG–3′) + Alt-ALR1 (5′– GACCCRGCYGGAGGDGGRG –3′), and LCO1490 + HCO2198 [26] were used to attempt to amplify a 608 bp and 708 bp fragment, respectively, of the 5′–end of COI of *Arrenurus* larvae and not their damselfly hosts. The thermocycler protocol for COI amplification was as follows: initial denaturation cycle at 94PC for 3 min, followed by 45 cycles of 94PC for 45 s, primer annealing at 45PC for 45 s, 72PC for 1 min, and a final extension at 72PC for 5 min.

Amplified products and negative controls were visualized on 1% agarose electrophoresis gels, and purified using pre-cast E-Gel CloneWell 0.8% SYBR Safe agarose gels (Invitrogen, Carlsbad, CA, USA) following Gibson et al. [27]. Sequencing reactions followed the protocol of Knee et al. [28], and sequencing was performed at the Agriculture & Agri-Food Canada, Eastern Cereal and Oilseed Research Centre Core Sequencing Facility (Ottawa, ON, Canada).

Sequence chromatograms were edited and contiguous sequences were assembled using Sequencher v4.7 (Gene Codes Corp., Ann Arbor, MI, USA). COI sequences were aligned manually in Mesquite v2.74 [29] according to the translated amino acid sequence. Sequences have been submitted to GenBank (KF880845–KF880906). Homologous sequences from eight *Arrenurus* sp. individuals collected from *Nehalennia gracilis* and *N. irene* damselflies [21] were included in the COI alignment, and seven water mite species were selected from GenBank to serve as outgroups (AB530314, JX838402, JX836526, JX835088, JN018105, JN018109, AB530311).

Pairwise distances were calculated using neighbour-joining analysis with the uncorrected (“p”) model in PAUP* v4.0b10 [30]. Phylogenetic analysis of the COI dataset was performed using Bayesian inference (BI) in MrBayes v3.1.2 [31,32]. The best-fit model of molecular evolution was determined to be GTR + I + G, using MrModeltest v2.3 [33]. Bayesian analysis was performed in MrBayes with a Markov Chain Monte Carlo (MCMC) method, two independent runs, with nucmodel = 4by4, N_st_ = 6, rates = invgamma, samplefreq = 1000, four chains = one cold and three heated, 10 million generations. In Mesquite, the remaining trees, excluding the burn-in (100), were used to generate a majority-rule consensus tree displaying the posterior probability supports for each node. Bayesian analysis was performed using the Cyberinfrastructure for Phylogenetic Research (CIPRES) portal [34]. In TNT v1.1 [35] node support was assessed using jackknife resampling with 36% of characters removed and 1000 replicates, using a heuristic search with tree bisection-reconnection (TBR) branch swapping and 1000 random addition sequence replicates.

### Statistical measures of mite richness and composition

We used the Bray-Curtis dissimilarity index to determine whether water mite species richness and composition differed more between or within host species pairs in R using vegdist in the package VEGAN [36].

## Authors’ contributions

JJM MRF and WK co-wrote the manuscript. JJM and MRF conceived of the study and design. JJM collected the data. JJM and MRF performed the statistical analyses. WK performed the molecular work and analyses. All authors read and approved the final manuscript.
